# Protein Loop Modeling Using a New Hybrid Energy Function and Its Application to Modeling in Inaccurate Structural Environments

**DOI:** 10.1371/journal.pone.0113811

**Published:** 2014-11-24

**Authors:** Hahnbeom Park, Gyu Rie Lee, Lim Heo, Chaok Seok

**Affiliations:** Department of Chemistry, Seoul National University, Seoul, Republic of Korea; University of Michigan, United States of America

## Abstract

Protein loop modeling is a tool for predicting protein local structures of particular interest, providing opportunities for applications involving protein structure prediction and *de novo* protein design. Until recently, the majority of loop modeling methods have been developed and tested by reconstructing loops in frameworks of experimentally resolved structures. In many practical applications, however, the protein loops to be modeled are located in inaccurate structural environments. These include loops in model structures, low-resolution experimental structures, or experimental structures of different functional forms. Accordingly, discrepancies in the accuracy of the structural environment assumed in development of the method and that in practical applications present additional challenges to modern loop modeling methods. This study demonstrates a new strategy for employing a hybrid energy function combining physics-based and knowledge-based components to help tackle this challenge. The hybrid energy function is designed to combine the strengths of each energy component, simultaneously maintaining accurate loop structure prediction in a high-resolution framework structure and tolerating minor environmental errors in low-resolution structures. A loop modeling method based on global optimization of this new energy function is tested on loop targets situated in different levels of environmental errors, ranging from experimental structures to structures perturbed in backbone as well as side chains and template-based model structures. The new method performs comparably to force field-based approaches in loop reconstruction in crystal structures and better in loop prediction in inaccurate framework structures. This result suggests that higher-accuracy predictions would be possible for a broader range of applications. The web server for this method is available at http://galaxy.seoklab.org/loop with the PS2 option for the scoring function.

## Introduction

Loops are often involved in the functional regions of proteins [1]–[4]. An accurate method for predicting the three-dimensional loop structure can be an invaluable tool for *de novo* design of novel proteins or small molecules involving protein loops in the binding interfaces. However, due to large variations in loop sequences, homologous proteins often lack structural information in the loop region, thereby making template-based approaches difficult to apply.

A number of *ab initio* loop modeling methods have been reported to show successful results in reconstructing loops in high-resolution crystal structures [5]–[11]. However, loops of interest for practical applications are often situated in non-ideal conditions. For example, loops may need to be modeled in low-resolution crystal structures, ensembles of NMR structures, or homology models for applications to molecular replacement [12], structure-based drug design [13], or antibody design [14]. Therefore, in the next era of loop modeling, achieving atomic-accuracy predictions in framework structures with errors will become a new challenge.

A strategy employed in recent studies to tackle this issue was to extend the sampling region to the environment of the target loop. When tested on high-resolution crystal structures with deliberately perturbed side chains around the loop, simultaneous sampling of the loop and surrounding side chains resulted in a performance comparable to that of loop reconstruction in the crystal environment [15], [16]. Nevertheless, there is a chance that this strategy is successful because the backbone structures are fixed at the crystal structures. It still needs to be shown whether expanding the sampling region by itself can be successfully applied to the modeling of loops involving larger environmental errors.

In this study, a complementary strategy is suggested that employs an energy function adequate for scoring model structures in an inaccurate environment. A hybrid energy function that combines physics-based components and knowledge-based components is designed to take advantage of the strengths of the two types of scoring functions: the physics-based energy terms help to locate precise structures near the native structure, and the knowledge-based terms tend to smooth the free energy surface so that environmental inaccuracy can be tolerated. This new strategy is demonstrated to provide high-accuracy predictions for loops in unreliable structural environments.

The new loop modeling method, called GalaxyLoop-PS2, was tested on loop sets in environments with a range of errors, from crystal structures to perturbed structures in both backbone and side chains and template-based model structures. The test results are encouraging when compared to state-of-the-art methods based on molecular mechanics force fields [10], [15], showing comparable performance both in the crystal environments and in inaccurate environments even when no extended sampling is attempted. A free web service for GalaxyLoop-PS2 is provided at http://galaxy.seoklab.org/loop with the PS2 option for the scoring function.

## Results and Discussion

### Loop modeling test sets with variable environmental accuracies

In order to estimate to what extent the environmental errors affect loop modeling accuracy, four types of loop modeling test sets are employed. Details are described in the Methods section. The first type of test sets consists of a total of 73 loops (20 8-residue loops and 20 12-residue loops for Set 1, and 33 12-residue loops for Set 2) in high-resolution X-ray crystal structure environments. The performance on this set corresponds to the maximum performance that can be obtained in the exact framework structure. The second type of test sets consists of 40 loop targets taken from Set 1, but the framework structures are deliberately perturbed in the side chains (taken from Sellers *et al.* [15]). This set is named as the side chain-perturbed set. The third type of test sets consists of the same 40 loop targets, but the overall structures, including the backbone, were perturbed by 2-ns molecular dynamics simulations to introduce thermal fluctuations. This set, built in this study, is named as the backbone-perturbed set. The last test set is comprised of 23 loops in more inaccurate environment of template-based models.

**Figure 1:**
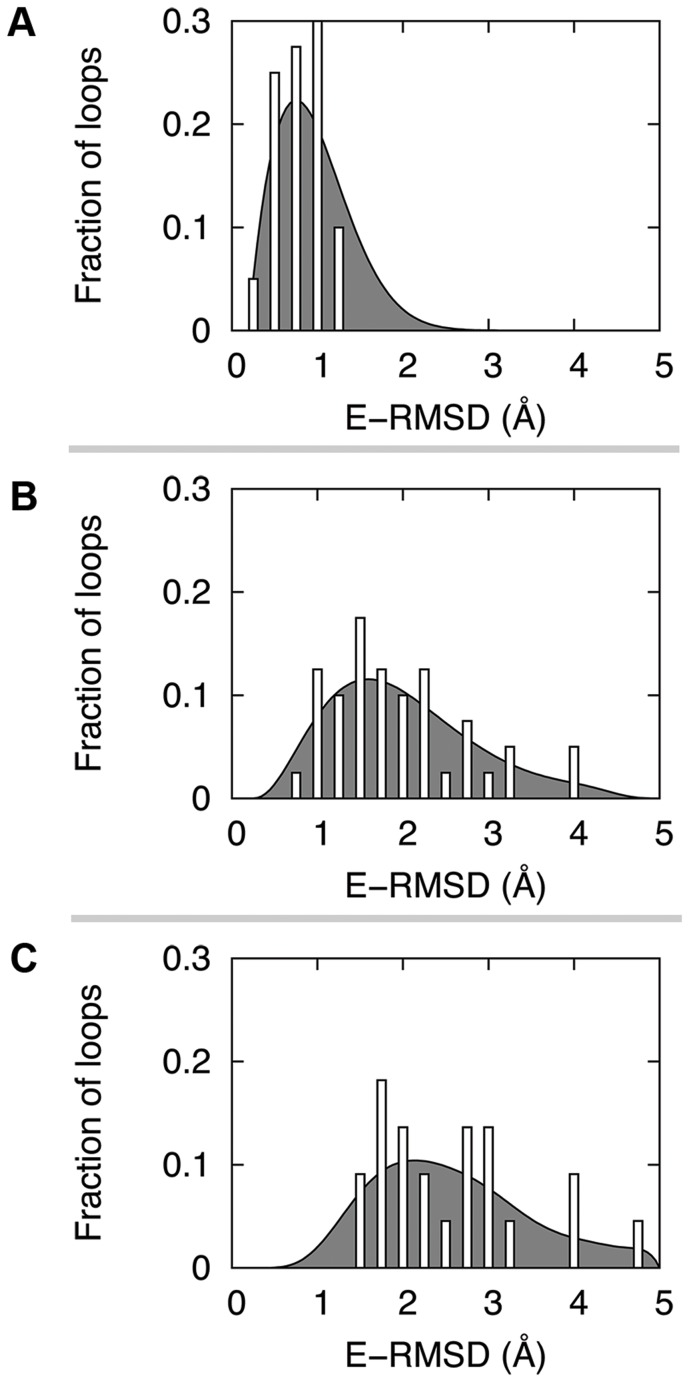
Distributions of environmental errors for the three types of test sets employed in the study. (A) for the test set of crystal structures with perturbed side chains, (B) for the crystal structures with both backbone and side chains perturbed, and (C) for the template-based models. The gray curve behind the histogram represents an interpolation. The average E-RMSD values are 0.9 Å, 2.1 Å, and 2.8 Å for the side chain-perturbed set (A), the backbone-perturbed set (B), and the template-based model set (C), respectively. E-RMSD represents the all-atom RMSD of environment residues for which any atoms are within 10 Å from any loop C_β_ atoms.

The distributions of environmental accuracies of the test sets are shown in **Figure 1**. Throughout the article, the deviation of the environmental structure of a loop from the experimental structure is measured by the all-atom root-mean-square deviation (RMSD) of the environment (E-RMSD), where the environment is defined as the set of residues with any atom within 10 Å from any loop C_β_ atoms. The E-RMSD is then calculated after superimposing the environmental structure onto the corresponding experimental structure. All RMSD values in this paper were calculated considering that flipping of symmetric side chains produces equivalent structures. As **Figure 1** shows, the E-RMSD increases from the side-chain perturbed set to backbone-perturbed set and template-based model set with averages of 0.9 Å, 2.1 Å, and 2.8 Å, respectively.

### Loop reconstruction in the framework of the crystal structure

The new loop modeling method introduced in this study (GalaxyLoop-PS2) is compared with the method developed previously for template-based model refinement (GalaxyLoop-PS1, [17]) and other state-of-the-art methods, HLP, HLP-SS [10], [15], Rosetta-KIC [16], and Next-generation KIC (NGK) [18]. The energy of GalaxyLoop-PS1 was optimized for application to the refinement of template-based models, while that of GalaxyLoop-PS2 was developed for higher performance in near-native environments as well, as explained in the Methods section. HLP and HLP-SS use a physics-based energy function with an implicit solvation model (OPLS-AA [19]–[21] and SGB [22], [23]). Rosetta-KIC and NGK use the Rosetta full-atom energy. While the Rosetta energy function has a hybrid form like GalaxyLoop-PS2, the main difference lies in the extent of physics-based and knowledge-based energy terms used. In the Rosetta energy function, the knowledge-based terms mainly serve to describe short-range interactions and interactions between charged amino acids, and the physics-based part does not contain Coulomb electrostatic energy. In GalaxyLoop-PS2, more complete energy terms are used for both physics-based and knowledge-based terms to combine the strengths of the two types of energy functions. In addition, a higher-level solvation free energy function is used in GalaxyLoop-PS2 (See Methods for details). The two GalaxyLoop methods and HLP perform sampling only of the loop regions, while HLP-SS, Rosetta-KIC, and NGK extend sampling to surrounding residues.

In the test of crystal structure reconstruction, GalaxyLoop-PS2 produces results superior to GalaxyLoop-PS1 and comparable to HLP, HLP-SS, Rosetta-KIC, and NGK as summarized in **Table 1**. Results for individual loop targets are reported in **Tables S1, S2, and S3**. It is notable that with GalaxyLoop-PS2, an average main chain RMSD of less than 1 Å is obtained for the 8-residue test set. HLP shows better results than the others for 12-residue loops of Set 1 (see **Table 1**), and the differences are mainly in the targets containing *cis*-proline residues (1cs6 and 1f46). GalaxyLoop-PS2 performs worse than another state-of-the-art method, ICMFF, which was tested on the 8-residue and 12-residue loops of Set 1 in the crystal environment with average RMSDs of 0.5 Å and 1.1 Å, respectively [11].

**Table 1:** Comparison of loop modeling results by the average RMSD of main chain atoms (N, C_α_, C, and O) of loops in angstroms (Å) on test sets of varying environmental accuracies measured by E-RMSD.

Framework	Loop set (No. residue)	E-RMSD (Å)	Loop Sampling1)			Extended Sampling1)		
			GalaxyLoop		HLP2)	HLP-SS2)	Rosetta-KIC3)	NGK4)
			PS2	PS1				
Crystal structure	Set 1 (8)5)	0±0	0.9±0.7	1.3±0.8	1.2±1.5	1.4±1.2	-	0.5±0.3
	Set 1 (12)6)	0±0	1.6±1.3	2.4±1.3	1.2±1.2	1.4±1.4	1.9±1.9	1.7±1.8
	Set 2 (12)7)	0±0	2.5±2.0	3.2±1.9	-	-	2.2±2.1	2.0±2.3
Side chain-perturbed	Set 1 (8)5)	0.9±0.3	1.3±0.9	1.8±1.5	2.4±1.6	1.3±1.5	-	0.5±0.3
crystal structure	Set 1 (12)6)	1.0±0.2	2.1±1.6	3.0±1.4	2.6±1.9	1.7±1.4	1.6±1.4	1.7±1.8
Backbone-perturbed	Set 1 (8)5)	1.9±0.6	2.0±1.8	2.2±1.5	-	-	-	2.1±1.8
crystal structure	Set 1 (12)6)	2.2±0.9	2.1±1.4	3.2±1.4	-	-	-	2.3±2.0

Standard deviations are also reported.

1) Loop sampling methods sample only the loop region, while extended sampling methods sample surrounding side chains in addition to the loop.

2) Taken from Sellers *et al.* [15].

3) Taken from Mandell *et al.* [16].

4) Results of the best-score models out of 500 models sampled for each target following the protocol provided by Stein *et al.* [18] with Rosetta v3.5.

The results for the crystal structure set and the side chain-perturbed set are the same for NGK because extended sampling of loop environment was used for both sets.

5) Loop sets taken from Jacobson *et al.* [10]. See **Tables S1** and **S2** for the list of loops.

6) Loop sets from Zhu *et al.* [34]. See **Tables S1** and **S2** for the list of loops.

7) Loop set from Fiser *et al.* [1]. See **Tables S3** for the list of loops.

### Loop modeling in the framework of side chain-perturbed crystal structures

This section presents how perturbations to the experimental framework structures affect loop prediction accuracy. As the purpose of this study is to assess the performance of the energy function in inaccurate environments, it is again noted that no further sampling beyond the loop region was attempted.

The first test set employed for this purpose is the set of crystal structures with perturbed side chain structures taken from Sellers *et al.* [15]. Interestingly, the performance of GalaxyLoop-PS2 on this set is not greatly affected by imperfect neighboring side chains, as can be seen from **Table 1**. Results for individual targets are listed in **Tables S4 and S5**. The increases in average main chain RMSDs from those of the crystal structure reconstruction tests are 0.4 Å (from 0.9 to 1.3 Å) and 0.5 Å (from 1.6 to 2.1 Å) for 8-residue and 12-residue loops, respectively. Sub-angstrom models were obtained in 50% and 20% of 8- and 12-residue loop targets, respectively. HLP, which utilizes a molecular mechanics energy function, performs worse in this test than in the crystal structure reconstruction test, with an increase in average RMSDs by 1.2 Å (from 1.2 to 2.4 Å) and 1.4 Å (from 1.2 to 2.6 Å) for 8- and 12-residue loops, respectively.

**Figure 2:**
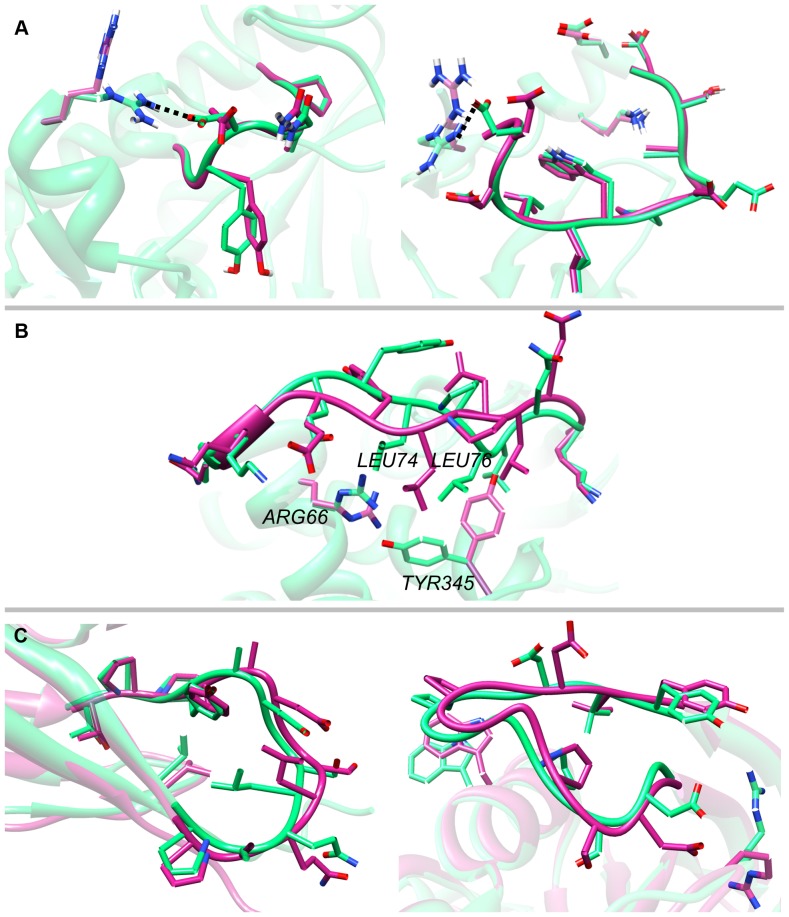
Examples of loops modeled in inaccurate environmental structures. In all panels, the crystal structures are colored in green and the models in magenta. Framework structures are shown transparent for clarity. (A) Two examples of tolerating errors in surrounding side chains, 1oyc (left; RMSD = 0.4 Å) and 1c5e (right; RMSD = 0.5 Å). The loop-framework salt bridges in the crystal structures are indicated with black dotted lines. High-accuracy modeling is possible even though the salt bridges cannot be recovered owing to the perturbed arginine orientations in the framework. (B) An example of unsuccessful modeling in the framework of perturbed side-chains, 1oth (RMSD = 2.3 Å), showing the necessity of additional sampling. The perturbed Arg66 and Tyr345 side chains (magenta) would clash with the two leucine residues in the loop if the crystal loop structure were to be placed. (C) Two examples of tolerating additional backbone errors, 1my7 (left; RMSD = 1.0 Å) and 1cb0 (right; RMSD = 0.9 Å). The overall backbone trace and key side-chain interactions are well reproduced.

The reason for the large discrepancy between the results of the two methods may be better understood by examining two examples (1oyc and 1c5e) illustrated in **Figure 2A**. The lowest-energy models generated by GalaxyLoop-PS2 have RMSD = 0.4 Å and 0.5 Å for 1oyc and 1c5e, respectively. However, when physics-based energy alone is used, the loops cannot be modeled with high accuracy, because the salt bridge between the loop and framework cannot be recovered due to the perturbed arginine side-chain structure in the environment. The loop modeling accuracy of HLP is RMSD = 2.2 Å and 1.8 Å for 1oyc and 1c5e, respectively. These examples demonstrate the high sensitivity of force field-based methods to small environment errors (E-RMSD = 0.9 Å and 0.7 Å for 1oyc and 1c5e, respectively). Similar salt bridge problems were identified in 8 out of the 40 loop targets. Several other sensitive cases could also be related to the strong dependence of electrostatic interactions to short-range local geometry. The sensitivity may also be related to the Generalized Born (GB) solvation model, which tends to over-stabilize salt bridge interactions [24]–[26]. Although the energy of GalaxyLoop-PS2 employs a GB solvation model, knowledge-based components, such as dipolar-DFIRE, appear to complement the sensitivity of the physics-based electrostatic energy function to the accuracy of local geometry.

When compared to methods that employ additional sampling of neighboring side chains (HLP-SS, Rosetta-KIC, and NGK in **Table 1**), GalaxyLoop-PS2 shows slightly worse loop modeling accuracies. The cases in which GalaxyLoop-PS2 failed to model accurately can be easily understood, such as the cases in which the perturbed side chain conformations do not allow native-like loop conformations owing to steric clashes, as illustrated in **Figure 2B** for 1oth. Such loops can be modeled more accurately only when the surrounding residues are sampled together.

### Loop modeling in the framework of backbone-perturbed crystal structures

To examine the performance of GalaxyLoop-PS2 in more difficult situations, loops were modeled for the same set of proteins (Set 1) but with further deviations in both backbone and side chain structures from the crystal structures. Environment error introduced by distorting ‘neighboring’ regions including backbone was shown to decrease loop modeling accuracy in previous works [1], [27], [28]. In this study, ‘global’ structure is perturbed to mimic actual situations of loop modeling in globally inaccurate frameworks. The performance of GalaxyLoop-PS2 on this set is compared to that of GalaxyLoop-PS1 run in this study and that of NGK run with the protocol provided by Stein *et al* [18]. The loop environments of backbone-perturbed set are more inaccurate compared to the side chain-perturbed set, with increases in E-RMSD by 1 Å (from 1 Å to 2 Å), as shown in **Table 1**. In addition, the loop anchor positions, which can affect prediction accuracy greatly [17], are also perturbed from the original structure. However, increase in the average RMSD of the loop models is smaller than that in environment. The average RMSD remains the same at 2.1 Å for 12-residue loops and increases by 0.7 Å (from 1.3 Å to 2.0 Å) for 8-residue loops when compared to those obtained for side-chain perturbed set. GalaxyLoop-PS2 performs comparably to NGK on this backbone-perturbed set although it does not involve extended sampling of environment structures. Detailed results on the individual targets are provided in **Tables S4 and S5**.

It must be noted that the RMSD value of a loop structure in an inaccurate environment is increased by the environmental inaccuracy as well as by the inaccuracy of the loop structure itself. For example, even a loop structure very close to the native structure is not guaranteed to have an RMSD close to 0, because the RMSD is calculated after structural superposition of the inaccurate environmental structure on the crystal structure.

The backbone trace of the loop and key side chain interactions can be predicted reasonably well, as illustrated for two examples in **Figure 2C**. These specific examples show prediction results with high accuracy (with loop RMSDs of 1.0 Å and 0.9 Å), while tolerating environments with much larger error (E-RMSD of 4.0 Å and 2.7 Å). Alongside the environment backbone, some perturbed side chains that can affect interactions with loop atoms, such as adjacent arginine residues involved in salt bridges, have been tolerated, similar to the cases observed in the side chain-perturbed set. On the other hand, targets that show greater failures compared to the previous tests were generally associated with large environmental perturbations that would cause steric clashes in native-like loop structures. Compared to GalaxyLoop-PS1, GalaxyLoop-PS2 still performs better on this set, although the gap between the two methods becomes smaller than on the previous sets. This can be explained by the fact that the energy function used for GalaxyLoop-PS1 was trained on a set of loops in more inaccurate environment structures in template-based models.

### Loop modeling in the framework of template-based models

An explorative test of loop modeling in template-based models was tried to test the performance of GalaxyLoop-PS2 in more inaccurate environments. A set of 23 loop modeling targets were constructed from the HOMSTRAD set [29] using template-based models generated with MODELLER 9.6 [30]. The E-RMSD of this set ranges from 1.6 Å to 5.3 Å, with an average of 2.8 Å. The prediction results are summarized in **Table 2**, and details are reported in **Table S6**. Before discussing the results, it is worth pointing out that, similar to the case of the backbone-perturbed set, errors from structural superposition of the inaccurate environment can be embedded in the calculated loop RMSD.

**Table 2:** Comparison of loop modeling results on the test set of template-based models.

Framework	Loop set (No. residue)	E-RMSD (Å)	Loop RMSD (Å)				
			GalaxyLoop		MODEL-LER1)	ModLoop2)	NGK3)
			PS2	PS1			
Template-based model	TBM set4) (6–11)	3.0±1.3	3.7±1.4	3.9±1.6	4.2±1.9	4.0±1.7	3.9±1.5

The average RMSD and its standard deviation are reported in Å. The Loop RMSD is calculated as the root-mean-square deviation of the main-chain atoms N, C_α_, C, and O.

1) Loop conformations generated by MODELLER [30].

2) Loop conformations generated by loop refinement using ModLoop of MODELLER [1], [27].

3) Results of the best-score models sampled by Next-generation KIC (NGK) using the protocol provided by Stein *et al.* [18].

500 models were generated for each target as in Stein *et al.* The Rosetta program v3.5 was used.

4) Loop set constructed in this study. See **Table S7** for the list of loops.

**Figure 3:**
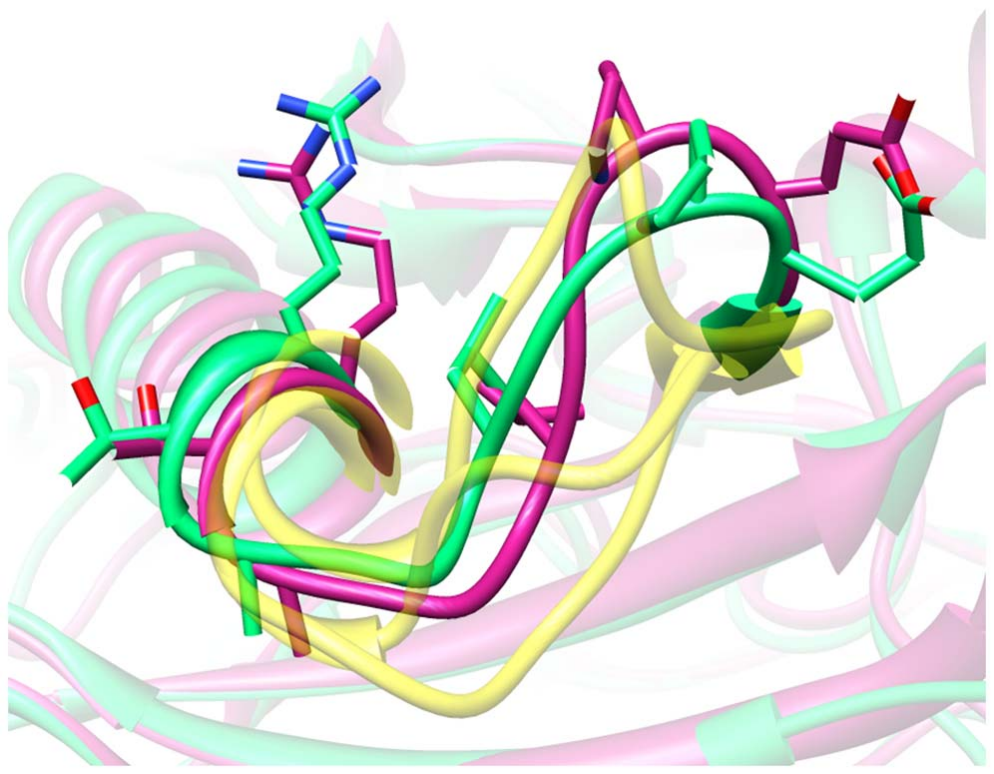
A successful example of loop modeling in the framework of a template-based model. The crystal structure is colored in green and the model in magenta (1avk, RMSD = 1.5 Å). Framework structures are shown transparent for clarity. Loops of three templates (used for template-based modeling) are shown with yellow transparent ribbons for comparison.

To briefly state the results, loops in the template-based model set were predicted with RMSD <3 Å in 7 out of 23 cases and <2 Å in 3 cases by GalaxyLoop-PS2. On average, the loop structures predicted by GalaxyLoop-PS2 (average RMSD of 3.7 Å) and GalaxyLoop-PS1 (average RMSD of 3.9 Å) are more accurate than the loops in the template-based models generated by MODELLER (average RMSD of 4.2 Å), the loop models after loop refinement using ModLoop (average RMSD of 4.0 Å) [1], [27], [30]. In addition, the results are comparable to those of NGK which carries out extended optimization of environment (average RMSD of 3.9 Å). One of the outstanding examples is illustrated in **Figure 3**, in which even side chain orientations can be modeled accurately. *Ab initio* loop modeling is necessary for this target, since the corresponding loop structures of the three template proteins used for template-based modeling (yellow ribbons in the figure) do not contain useful structure information.

It must be noted that the absolute degree of improvement achieved by the *ab initio* loop modeling is rather limited when applied to the case of large environmental errors such as template-based models. This implies that the current approach of using a new energy function insensitive to environmental errors is insufficient for improving template-based models to atomic accuracy and that refinement of the surroundings by extending the sampling region is required.

### Sampling performance of GalaxyLoop-PS2

Thus far, the lowest-energy model structures were examined in the above analysis. The GalaxyLoop methods generate 30–50 models, so it would be worthwhile to examine the ensemble of generated structures to assess the sampling performance. The quality of such a conformational ensemble can also be important for applications such as ligand docking on ensemble structures [31]–[33].

Overall, a majority of the loop ensembles generated by GalaxyLoop-PS2 contain models with a main chain RMSD <2 Å, even in inaccurate environments. For the side chain-perturbed sets, at least one model is sampled within 2 Å for 17 out of 20 8-residue loops and 16 out of 20 12-residue loops, as shown in **Table 3**. For the backbone-perturbed sets, this criterion is satisfied in 16 out of 20 cases for both 8- and 12-residue loops. As the conformational ensembles generated for the test loops contain native-like loop conformations in a majority of the cases, the current loop modeling method may be applicable to various practical applications that may utilize an ensemble of loop conformations.

**Table 3:** Sampling results of GalaxyLoop-PS2 on the three test sets.

Framework	Set (No. loop residue)	No. loops	No. loop sampled within1)		
			<1.0 Å	<1.5 Å	<2.0 Å
Crystal structure	Set 1 (8)	20	19	20	20
	Set 1 (12)	20	13	16	19
	Set 2 (12)	33	20	27	28
Side chain-perturbed	Set 1 (8)	20	14	16	17
	Set 1 (12)	20	8	16	16
Backbone-perturbed	Set 1 (8)	20	8	13	16
	Set 1 (12)	20	6	14	16

1) Number of loop targets for which at least one structure among the 30 loop conformations (or 50 conformations for 12-residue loops) in the final CSA bank is within a given RMSD value.

### Comparison of the hybrid energy with the physics-based and knowledge-based energy

One assumption underlying the design of the hybrid energy function in this study is that the advantages of the physics-based and knowledge-based energy terms can be synergized by combining the energy terms. To confirm this assumption, additional tests were performed on two energy functions constructed by taking only knowledge-based terms (with additional bonded energy terms to maintain proper local geometry) and by taking only physics-based terms from the hybrid energy function. When tested on the 12-residue loop targets of Set 1, the average RMSDs for the crystal, side chain-perturbed, backbone-perturbed, and template-based model sets are 1.7 Å, 2.3 Å, and 2.0 Å, respectively, for the knowledge-based energy, and 2.1 Å, 3.0 Å, and 2.8 Å, respectively, for the physics-based energy. For the same test sets, the hybrid energy gives 1.6 Å, 2.1 Å, and 2.1 Å. Detailed results on the individual targets are reported in **Table S7**. The hybrid energy function shows superior results to both the physics-based and knowledge-based energy functions, as anticipated, except in the case of a backbone-perturbed set, in which the knowledge-based energy shows slightly better performance than the hybrid energy by 0.1 Å. The excellent performance of the knowledge-based energy function on the backbone-perturbed set may be due to the fact that the backbone-perturbed framework structures represent a rather realistic thermal ensemble that can be captured by the smooth landscape of the knowledge-based energy.

It must be pointed out that the physics-based energy function performs poorly on the crystal framework test set compared to HLP, which is also based on similar energy terms. This may be because the current molecular mechanics energy uses polar hydrogen topology rather than an all-atom representation and a more approximate GB method called FACTS for computational efficiency. It is non-trivial to explain the lower performance of the physics-based part than the knowledge-based part in the crystal environment, but it is noted that the knowledge-based energy is actually combined with the bonded energy terms of the physics-based part. Loop modeling in a full atom representation that is more physically realistic will be pursued in the future.

It is suggested that the new hybrid energy function can be combined with an extended sampling of the surroundings. It is believed that such efforts to extend the applicable range of the current loop modeling techniques must be continued to solve various practical problems, such as structure-based drug design and experimental structure determination.

### Computational cost

The average computation time for the 12-residue loops of Set 1 and Set 2 is 92 CPU hours on 2.4-GHz Intel Xeon processors. Each job takes approximately 4 hours when run on 24 CPUs in parallel. The computation time could be reduced (down to 82 CPU hours) by using a smaller size of CSA bank (*N* = 30 instead of *N* = 50) with slight decrease in the prediction accuracy (average loop RMSD of 2.1 Å, 2.2 Å, and 2.6 Å for the 12-residue loop set in three different environments, respectively, compared to 1.6 Å, 2.1 Å, and 2.1 Å with *N* = 50. See **Table S8** for details.). Average computation time for the same set is 182 h for NGK when 500 models are generated for each target. This can be compared to the reported computation times of 320 h for Rosetta-KIC (to generate 1000 models), 260 h for HLP-SS, 55 h for ICMFF (for each run, results after 5 runs were reported in Arnautova et al., 2011), and 29 h (N = 30) and 95 h (*N* = 50) for GalaxyLoop-PS1.

## Methods

### Loop modeling test sets

Four types of loop modeling test sets with different degrees of error in the framework structure were employed. The first type consists of two subsets of crystal structure frameworks, one including 20 8-residue loops from Jacobson *et al.* [10] and 20 12-residue loops from Zhu *et al.* [34] (called Set 1) and another composed of 33 12-residue loops from Fiser *et al.* [1] (called Set 2). The second type consists of the same loop targets as Set 1 of the first type, but with perturbed side chain structures for the residues surrounding the loops as generated by Sellers *et al.* [15] (downloaded from http://www.jacobsonlab.org/decoy.htm). The third type consists of the same loop targets as the side chain-perturbed set, but the framework structures are perturbed in the overall structure, including the backbone. This set is called the backbone-perturbed set.

The backbone-perturbed set was prepared by performing 2-ns molecular dynamics (MD) simulations at 300 K, starting from the energy-minimized crystal structures using the AMBER12 package [35]. The AMBER99SB force field [36] and the Generalized Born/Surface Area (GB/SA) implicit solvation model [37], [38] were used. Considering that MD simulations generate thermally accessible conformational fluctuations, the tests on the backbone-perturbed set may be regarded as loop modeling tests in framework structures from thermal ensembles.

The fourth type of test set consists of loop targets in template-based models. The protein targets for this set were collected from the HOMSTRAD set [29]. Template-based models for the protein targets were generated using MODELLER 9.6 [30] with templates and multiple sequence alignments taken from the SALIGN benchmark study [39], [40] (downloaded from http://salilab.org/projects/salign). Only those targets for which the template-based models have GDT-TS [41] between 70 and 90 were considered. The target loop regions were selected with the model consensus method for detecting unreliably modeled regions [42]. Loops involved in interactions with other protein chains or ligand molecules, those in crystal contacts with other subunits, and those in NMR structures which show large fluctuations were not considered. This resulted in 23 loop modeling targets. The backbone-perturbed set and template-based model set can be downloaded from http://galaxy.seoklab.org/suppl/ps2.html.

### Energy function

In GalaxyLoop-PS2, the energy function is described by a sum of physics-based energy terms and knowledge-based energy terms as follows:
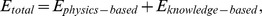


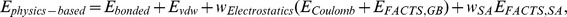


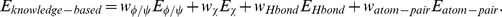
All key molecular interactions, such as short-range interactions, electrostatic interactions including solvation effect, and hydrophobic interactions, are included in both the physics-based and the knowledge-based energy terms. By maintaining completeness within each type of energy function as much as possible, it is anticipated that a weakness in any part of one type of energy can be compensated for by the corresponding term in another type of energy. The physics-based energy is based on the CHARMM22 force field [43] mapped onto a polar hydrogen topology with bonded energy (*E*_bonded_), van der Waals energy (*E*_vdW_), Coulomb potential energy (*E*_Coulomb_), and the FACTS GB/SA solvation free energy (*E*_FACTS,GB_ and *E*_FACTS,SA_) [44]. The knowledge-based energy contains torsion angle correction terms (*E*_φ/ψ_ for backbone torsion angles and *E*_χ_ for the side-chain torsion angles derived in this study) to recover statistical preferences in local structure, the hydrogen-bond energy developed by Kortemme *et al.* [45] (*E*_Hbond_) to describe short-range electrostatics, and knowledge-based, atom-pair potential dipolar-DFIRE [46] (*E*_atom-pair_) to describe both short-range and long-range interactions and hydrophobic interactions. In particular, *E*_φ/ψ_ serves to correct secondary structure biases due to imperfect parameter optimization, as in the empirical modifications to the backbone torsion terms of molecular mechanics force fields [36], [47]. Details on the FACTS solvation free energy and the torsion angle knowledge-based energy terms newly implemented in GalaxyLoop in this study are described in more detail in **Text S1**.

The weight parameters are set to (*w*_Electrostatics_, *w*_SA_, *w*_φ/ψ_, *w*_χ,_ *w*_Hbond_, *w*_atom-pair_) = (0.16, 0.05, 1.2, 1.0, 4.0, 12.0) by training on the 28 training loop targets introduced in the previous study, using a similar optimization method that employs decoy loop conformations [17]. In this work, only the performance of loop reconstruction in the crystal structure framework was optimized, without further training on loops in template-based models. First, a grid search for optimal weights was performed for the relative weight between the physics-based part and the knowledge-based part, while the initial weights within each type of energy function were fixed to achieve an overall balance. Individual weights were then tuned. The torsion angle correction term for the backbone (*E*_φ/ψ_) was derived after determining all other energy weights, and then all weight parameters were once again tuned, including the *E*_φ/ψ_ term. Contribution of each energy term was analyzed by examining variations of the energy value (**Table S9**) and energy-RMSD correlation (**Table S10**) for the training set decoy conformations as explained in detail in **Text S1**.

### Loop modeling protocol

The GalaxyLoop-PS2 loop modeling follows the conformational space annealing (CSA) [48] global optimization procedure as reported previously for GalaxyLoop-PS1 [17], [49]. A flowchart of the method is provided in **Figure S1** and details on each step of the procedure are given by Park and Seok [17]. A pool of a fixed number of loop structures (*N* = 30 for loops of <12 residues and 50 for loops of ≥12 residues), called ‘bank’, is evolved by generating trial conformations by mixing pool conformations, as in a genetic algorithm, and by updating the pool by comparing the energies and distances of the bank members and the trial conformations at each iteration step. The initial bank is generated by the fragment assembly with loop closure (FALC) loop sampling procedure [50], [51], and the tri-axial loop closure algorithm [52] is used to maintain the structural integrity of the loop after mixing the conformations. The diversity of the pool is gradually reduced with each iteration by using a control parameter called ‘*D*_cut_’ that sets a distance criterion for replacing old bank members with trial conformations. The number of conformations in the final bank is the same as that in the initial bank, and the energy minimum structure in the final bank is selected as the final model.

After GalaxyLoop-PS1, a new aspect introduced in the current development is that a more extensive side chain sampling is performed. Each trial loop conformation generated during global optimization is subjected to an additional side chain sampling by a maximum of three trials of side chain conformation exchanges with other bank members. The trial loop conformation is further refined by short MD simulation and local energy minimization. In addition, a larger bank size (*N* = 50) was used for the 12-residue loops to alleviate sampling problems for these longer loops, while *N* = 30 was used by Park and Seok, regardless of the loop length [17].

## Supporting Information

Figure S1 **Flowchart of the GalaxyLoop-PS2 protocol.** The overall procedure follows the conformational space annealing global optimization. The FALC (fragment assembly with loop closure) method is used for generating initial conformations. A pool of N conformations is generated and evolved while gradually reducing the Dcut parameter, which controls the conformational diversity of the pool. (Here, (*M, N*) = (10, 30) for loops <12 residues and (20, 50) for loops ≥12 residues.)
(TIF)

Table S1 **Loop reconstruction results for the 8-residue loop Set 1.**
(PDF)

Table S2 **Loop reconstruction results for the 12-residue loop Set 1.**
(PDF)

Table S3 **Loop reconstruction results for the 12-residue loop Set 2.**
(PDF)

Table S4 **Loop modeling results on the perturbed crystal structures for the 8-residue loop Set 1.**
(PDF)

Table S5 **Loop modeling results on the perturbed crystal structures for the 12-residue loop Set 1.**
(PDF)

Table S6 **RMSD results of the modeled loops for template-based models.**
(PDF)

Table S7 **Loop reconstruction results and modeling results on perturbed crystal structures for the 12-residue loop Set 1 using energy functions composed of either knowledge-based or physics-based energy components.**
(PDF)

Table S8 **Loop modeling results for the 12-residue loop Set 1 in three different environments with a smaller number of CSA bank size (** ***N*** **= 30 instead of** ***N*** **= 50).**
(PDF)

Table S9 **Contribution of each energy component.**
(PDF)

Table S10 **Correlation between energy and decoy loop RMSD calculated using different subsets of energy components.**
(PDF)

Text S1 **Detailed information on Methods and Results.**
(PDF)
