# Supplementary figures and images for: Protein Loop Modeling Using a New Hybrid Energy Function and Its Application to Modeling in Inaccurate Structural Environments
Source: PLoS One. 2014 Nov 24;9(11):e113811. doi: 10.1371/journal.pone.0113811 (PMC4242723; doi:10.1371/journal.pone.0113811)

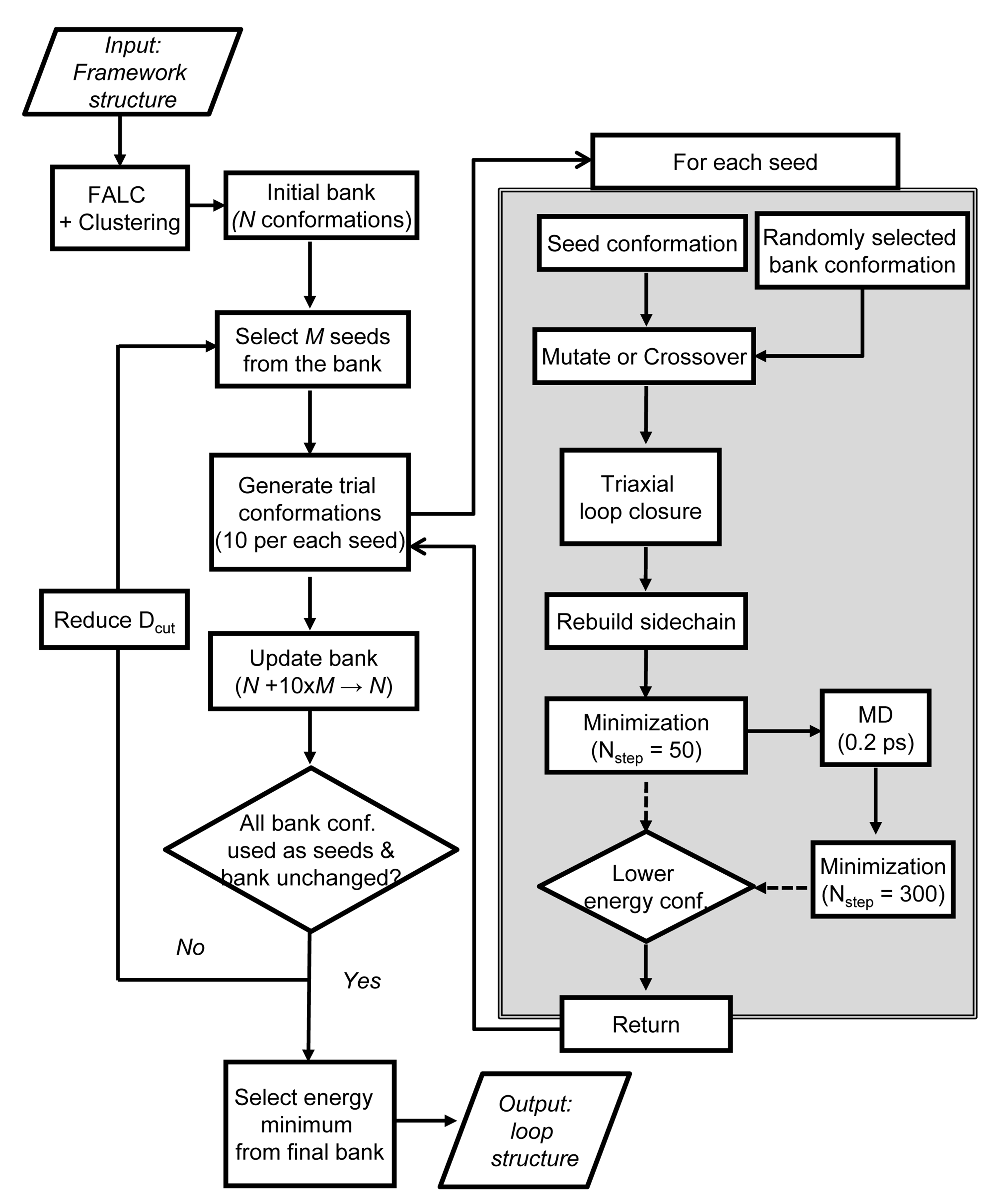

Supplement: Figure S1 — Flowchart of the GalaxyLoop-PS2 protocol. The overall procedure follows the conformational space annealing global optimization. The FALC (fragment assembly with loop closure) method is used for generating initial conformations. A pool of N conformations is generated and evolved while gradually reducing the Dcut parameter, which controls the conformational diversity of the pool. (Here, (M, N) = (10, 30) for loops <12 residues and (20, 50) for loops ≥12 residues.) (TIF) [file pone.0113811.s001.tif]
