# Supplementary material for: Protein Loop Modeling Using a New Hybrid Energy Function and Its Application to Modeling in Inaccurate Structural Environments
Source: PLoS One. 2014 Nov 24;9(11):e113811. doi: 10.1371/journal.pone.0113811 (PMC4242723; doi:10.1371/journal.pone.0113811)
Supplement: Table S1 — Loop reconstruction results for the 8-residue loop Set 1. (PDF) [file pone.0113811.s002.pdf]

Table S1. Loop reconstruction results for the 8-residue loop Set 1.

PDB ID	Loop range	Native framework, RMSD (Å)[1]					
		HLP[2]	HLP-SS[2]	NGK[3]	Galaxy PS1[4]	Galaxy PS2[5]	Best sampled (rank)[6]
135l	84–91	0.4	2.6	0.3	1.5	0.5	0.5 (1)
1alc	34–41	5.9	4.5	0.3	0.3	0.4	0.4 (1)
1btl	50–57	0.3	1.6	0.4	1.5	1.2	0.8 (2)
1cex	73–80	3.9	3.7	0.3	0.7	1.1	0.5 (6)
1clc	313–320	0.3	3.0	0.4	0.4	0.3	0.3 (1)
1ddt	127–134	1.0	0.9	1.0	1.1	1.0	1.0 (1)
1ezm	92–99	0.4	0.5	0.3	2.3	0.6	0.4 (2)
1hfc	142–149	0.2	0.3	0.5	0.9	0.6	0.5 (4)
1iab	48–55	1.6	0.8	0.5	2.1	1.9	0.6 (4)
1ivd	413–420	0.9	1.1	0.8	3.5	2.9	0.8 (12)
1lst	101–108	0.6	0.7	0.5	0.6	0.5	0.4 (4)
1nar	192–199	0.6	1.5	1.3	2.6	2.0	1.0 (5)
1oyc	80–87	0.5	0.4	0.3	0.6	0.5	0.5 (3)
1prn	150–157	1.7	2.4	0.3	1.6	0.5	0.5 (1)
1sbp	107–114	3.3	0.3	0.3	0.5	0.3	0.3 (1)
1tml	187–194	0.3	1.4	0.5	1.6	0.9	0.5 (5)
2cmd	270–277	0.4	0.4	0.4	0.6	0.5	0.5 (5)
2exo	262–269	0.4	0.4	0.3	0.9	0.5	0.3 (4)
2sga	32–43	1.2	1.2	1.3	1.1	0.9	0.3 (3)
5p21	45–52	0.4	0.9	0.3	0.8	0.3	0.3 (1)
**Average**		**1.2**	**1.4**	**0.5**	**1.3**	**0.9**	**0.5 (3.3)**
**Std. dev.**		**1.5**	**1.2**	**0.3**	**0.8**	**0.7**	**0.2 (2.6)**

1) RMSD is calculated as the root-mean-square deviation of the main-chain atoms N, C_α, C, and O.

2) Taken from Sellers *et al.* [1]

3) Results of the best-score models sampled by Next-generation KIC (NGK) using the protocol provided by Stein *et al.* [2]. 500 models were generated for each target as in Stein *et al.* The Rosetta program v3.5 was used.

4) Results of the lowest-energy model structures obtained by GalaxyLoop-PS1

5) Results of the lowest-energy model structures obtained by GalaxyLoop-PS2

6) RMSDs of the lowest-RMSD model structures and their energy ranks in the final bank

[1] Sellers BD, Zhu K, Zhao S, Friesner RA, Jacobson MP (2008) Toward better refinement of comparative models: predicting loops in inexact environments. Proteins 72: 959-971.

[2] Stein A, Kortemme T (2013) Improvements to robotics-inspired conformational sampling in rosetta. PLoS One 8: e63090.
