# Supplementary material for: Protein Loop Modeling Using a New Hybrid Energy Function and Its Application to Modeling in Inaccurate Structural Environments
Source: PLoS One. 2014 Nov 24;9(11):e113811. doi: 10.1371/journal.pone.0113811 (PMC4242723; doi:10.1371/journal.pone.0113811)
Supplement: Table S3 — Loop reconstruction results for the 12-residue loop Set 2. (PDF) [file pone.0113811.s004.pdf]

Table S3. Loop reconstruction results for the 12-residue loop Set 2

PDB ID	Loop range	RMSD (Å)[1)]				
		Rosetta KIC[2)]	NGK[3)]	Galaxy PS1[4)]	Galaxy PS2[5)]	Best sampled (rank)[6)]
154l	153–164	3.3	3.4	3.6	3.6	2.0 (38)
1arp	201–212	0.5	0.3	0.8	0.4	0.4 (1)
1ctm	9–20	2.9	0.7	4.8	5.9	3.3 (4)
1dts	41–52	5.2	1.1	7.2	1.6	1.1 (4)
1eco	35–46	0.4	0.4	1.6	0.7	0.7 (1)
1ede	150–161	0.7	1.2	3.3	2.6	0.8 (50)
1ezm	122–133	2.7	2.7	5.1	0.3	039 (2)
1hfc	165–176	8.2	8.1	4.4	7.3	2.0 (5)
1msc*	365–376	*3.2*	5.7	4.5	4.6	1.4 (35)
1onc	23–34	0.5	8.6	1.8	2.4	1.0 (19)
1pbe	129–140	0.6	0.5	1.0	1.8	0.6 (2)
1pmy	77–88	2.6	0.5	0.7	0.5	0.5 (1)
1prn	15–26	6.6	6.5	4.0	6.1	4.0 (47)
1rcf	88–99	0.6	0.5	2.9	4.1	0.8 (11)
1rro	17–28	0.4	0.5	2.5	2.3	0.7 (11)
1srp	311–322	0.6	0.3	0.5	0.5	0.4 (3)
1tca	305–316	0.6	0.5	2.5	2.3	0.5 (21)
1thg	127–138	1.1	1.1	2.9	2.5	1.6 (12)
1thw	178–189	2.7	0.8	2.6	2.2	1.1 (5)
1tib	99–110	1.2	1.8	1.5	1.2	1.2 (1)
1tml	243–254	0.4	0.3	2.4	1.6	0.9 (9)
1xif	203–214	0.7	0.5	1.7	1.8	0.9 (5)
2cpl	145–156	0.2	0.2	5.2	5.8	1.0 (47)
2ebn	136–147	2.1	2.2	2.1	2.5	1.4 (6)
2exo	293–304	0.8	0.4	4.6	0.3	0.3 (1)
2pgd	361–372	5.1	0.6	2.0	3.0	0.6 (11)
2rn2	90–101	0.8	1.1	5.8	6.8	5.1 (46)
2sil	255–266	1.0	1.8	1.6	1.1	0.6 (6)
2tgi	48–59	3.1	2.0	1.6	2.1	0.8 (38)
3hsc	72–83	0.5	0.5	4.5	0.4	0.4 (1)
451c	16–27	5.8	6.5	9.0	0.8	0.8 (5)
4enl	372–383	3.6	2.9	5.1	0.6	0.6 (1)
4i1b	46–57	3.8	5.2	4.2	5.1	1.5 (17)
**Average**		**2.2**	**2.0**	**3.2**	**2.5**	**1.2 (14.4)**
**Std. dev.**		**2.1**	**2.3**	**1.9**	**2.0**	**1.1 (16.5)**

* For 1msc, crystal symmetry was considered by all methods except Rosetta KIC. Therefore, the reported average value is calculated for 32 targets, excluding 1msc.

1) RMSD is calculated as the root-mean-square deviation of the main-chain atoms N, C_α, C, and O.

2) Taken from Mandell *et al.* [1]

3) Results of the best-score models sampled by Next-generation KIC (NGK) using the protocol provided by Stein *et al*. [2]. 500 models were generated for each target as in Stein *et al*. The Rosetta program v3.5 was used.

4) Results of the lowest-energy model structures obtained by GalaxyLoop-PS1

5) Results of the lowest-energy model structures obtained by GalaxyLoop-PS2

6) RMSDs of the lowest-RMSD model structures and their energy ranks in the final bank

[1] Sellers BD, Zhu K, Zhao S, Friesner RA, Jacobson MP (2008) Toward better refinement of comparative models: predicting loops in inexact environments. Proteins 72: 959-971.

[2] Stein A, Kortemme T (2013) Improvements to robotics-inspired conformational sampling in rosetta. PLoS One 8: e63090.
