# Supplementary material for: Protein Loop Modeling Using a New Hybrid Energy Function and Its Application to Modeling in Inaccurate Structural Environments
Source: PLoS One. 2014 Nov 24;9(11):e113811. doi: 10.1371/journal.pone.0113811 (PMC4242723; doi:10.1371/journal.pone.0113811)
Supplement: Table S5 — Loop modeling results on the perturbed crystal structures for the 12-residue loop Set 1. (PDF) [file pone.0113811.s006.pdf]

Table S5. Loop modeling results on the perturbed crystal structures for the 12-residue loop Set 1.

PDB ID	Loop range	Side chain–perturbed set RMSD (Å)[1]								Backbone-perturbed set RMSD (Å)[1]				
		E-RMSD (Å)[2]	HLP[3]	HLP-SS[3]	Rosetta KIC[4]	NGK[5]	Galaxy PS1[6]	Galaxy PS2[7]	Best sampled (rank)[8]	E-RMSD (Å)[2]	NGK[5]	Galaxy PS1[6]	Galaxy PS2[7]	Best sampled (rank)[8]
1a8d	155–166	0.7	0.9	2.8	0.6	5.2	4.1	0.5	0.5 (1)	1.6	3.7	4.5	3.1	1.3 (2)
1arb	182–193	0.9	2.2	2.6	1.4	0.4	3.7	1.0	1.0 (7)	2.3	1.7	2.1	1.9	1.4 (31)
1bhe	121–132	0.8	1.0	0.7	0.7	0.4	1.0	2.0	0.8 (10)	1.2	1.7	2.3	3.5	2.8 (20)
1bn8	298–309	0.7	8.3	2.6	0.6	1.1	5.4	1.6	0.8 (14)	3.4	1.1	4.3	1.1	1.1 (1)
1c5e	82–93	0.7	1.8	1.7	0.4	0.4	2.7	0.5	0.5 (1)	1.5	1.2	2.2	1.5	0.8 (22)
1cb0	33–44	0.8	0.4	0.3	0.7	0.6	0.7	0.5	0.5 (1)	2.7	0.9	5.7	0.9	0.9 (2)
1cnv	188–199	0.7	3.2	3.3	2.1	2.0	4.4	3.2	2.7 (2)	3.0	6.3	6.4	6.5	6.2 (7)
1cs6	145–156	1.1	3.4	3.5	3.0	2.5	1.8	3.1	2.8 (7)	1.8	1.1	1.7	1.6	1.2 (10)
1dqz	209–220	1.2	1.2	0.6	2.6	0.6	2.2	1.0	1.0 (1)	1.9	7.5	1.5	3.3	1.8 (23)
1exm	291–302	0.8	4.0	0.5	0.9	1.0	2.4	1.2	1.2 (1)	1.5	1.1	3.0	1.3	1.1 (6)
1f46	64–75	0.9	3.8	1.1	2.3	2.1	1.5	1.5	1.4 (41)	1.6	2.6	4.5	3.8	2.3 (32)
1i7p	63–74	0.9	0.5	0.3	0.4	0.4	2.8	0.3	0.3 (1)	3.3	1.9	2.8	1.7	1.5 (4)
1m3s	68–79	1.1	5.3	5.6	5.6	6.4	5.8	5.9	4.7 (36)	1.5	3.2	4.3	2.7	2.7 (23)
1ms9	529–540	0.8	2.9	2.5	1.0	2.7	1.9	2.6	1.2 (13)	1.6	1.8	1.8	1.8	0.9 (37)
1my7	254–265	1.5	1.4	0.9	2.3	0.6	3.6	2.9	0.6 (2)	4.0	0.9	2.4	1.0	1.0 (1)
1oth	69–80	1.1	2.3	0.7	0.6	0.4	1.1	2.3	1.5 (6)	1.2	0.8	1.1	0.9	0.9 (8)
1oyc	203–214	1.2	2.8	1.2	3.9	0.4	3.3	1.4	1.2 (22)	2.5	0.7	2.7	1.2	1.2 (2)
1qlw	31–42	0.6	1.5	1.4	0.9	4.8	4.6	4.6	2.7 (23)	2.3	6.0	2.5	2.9	1.5 (43)
1t1d	127–138	1.3	0.6	1.0	0.8	0.7	3.3	1.0	1.0 (1)	4.0	1.3	2.5	1.5	1.2 (2)
2pia	30–41	1.1	3.7	0.5	0.9	0.8	4.4	5.5	1.0 (16)	1.2	0.7	4.5	0.7	0.7 (2)
**Average**		**1.0**	**2.6**	**1.7**	**1.6**	**1.7**	**3.0**	**2.1**	**1.4 (10.3)**	**2.2**	**2.3**	**3.2**	**2.1**	**1.6 (13.9)**
**Std. dev.**		**0.2**	**1.9**	**1.4**	**1.4**	**1.8**	**1.4**	**1.6**	**1.0 (11.7)**	**0.9**	**2.0**	**1.4**	**1.4**	**1.2 (13.3)**

1) RMSD is calculated as the root-mean-square deviation of the main-chain atoms N, C_α, C, and O.

2) The all-atom RMSD of the environment is as defined in Results and Discussion

3) Taken from Sellers *et al.* [1]

4) Taken from Mandell *et al.* [2]

5) Results of the best-score models sampled by Next-generation KIC (NGK) using the protocol provided by Stein *et al*. [3]. 500 models were generated for each as in Stein et al. The Rosetta program v3.5 was used.

6) Results of the lowest-energy model structures obtained by GalaxyLoop-PS1

7) Results of the lowest-energy model structures obtained by GalaxyLoop-PS2

8) RMSDs of the lowest-RMSD model structures and their energy ranks in the final bank

[1] Sellers BD, Zhu K, Zhao S, Friesner RA, Jacobson MP (2008) Toward better refinement of comparative models: predicting loops in inexact environments. Proteins 72: 959-971.

[2] Mandell DJ, Coutsias EA, Kortemme T (2009) Sub-angstrom accuracy in protein loop reconstruction by robotics-inspired conformational sampling. Nature Methods 6: 551-552.

[3] Stein A, Kortemme T (2013) Improvements to robotics-inspired conformational sampling in rosetta. PLoS One 8: e63090.
