# Supplementary material for: Protein Loop Modeling Using a New Hybrid Energy Function and Its Application to Modeling in Inaccurate Structural Environments
Source: PLoS One. 2014 Nov 24;9(11):e113811. doi: 10.1371/journal.pone.0113811 (PMC4242723; doi:10.1371/journal.pone.0113811)
Supplement: Table S6 — RMSD results of the modeled loops for template-based models. (PDF) [file pone.0113811.s007.pdf]

Table S6. RMSD results of the modeled loops for template-based models.

PDB ID	Loop region (length)	Model quality			Loop RMSD (Å)[4]							
		GDT-TS[1]	E-RMSD (Å)[2]	Anchor RMSD (Å)[3]	MODEL-LER[5]	PS-knowledge[6]	PS-Physics[7]	Mod-Loop[8]	NGK[9]	Galaxy PS1[10]	Galaxy PS2[11]	Best sampled (rank)[12]
1d2kA	349–354 (6)	83.2	1.6	0.9	2.5	4.0	3.9	2.0	1.9	4.0	5.7	1.7 (21)
1a49A	86–94 (9)	70.5	1.6	1.0	3.4	3.4	4.8	3.4	4.6	3.8	4.2	3.3 (14)
1gpb	261–271 (11)	89.3	1.8	1.5	9.6	5.2	5.6	1.9	4.3	4.4	3.0	1.9 (14)
1qu9A	93–98 (6)	89.7	1.8	2.7	7.4	6.3	5.8	8.7	7.0	6.6	6.3	6.3 (7)
1avk	20–29 (10)	86.6	1.9	1.9	2.9	3.6	3.8	3.1	1.2	1.2	1.5	1.3 (4)
3fib	40–47 (8)	88.5	2.0	0.7	2.3	2.4	2.7	4.1	2.8	2.8	2.6	2.1 (9)
1e0cA	53–60 (8)	82.4	2.2	2.1	3.5	6.5	3.3	3.7	3.8	6.7	5.9	2.9 (20)
1esl	40–48 (9)	84.2	2.1	1.4	2.4	2.6	3.0	3.5	3.0	3.2	2.4	2.2 (22)
1bucA	61–68 (8)	88.0	2.2	1.8	2.7	2.3	2.7	4.5	5.3	3.9	3.2	2.2 (17)
2oatA	347–353 (7)	80.6	2.3	1.6	5.3	1.9	5.0	3.6	2.7	3.0	5.0	2.3 (27)
1qdlB	59–67 (9)	88.2	2.3	1.6	2.1	2.8	2.4	4.8	4.2	2.7	2.8	2.4 (16)
2polA	23–32 (10)	77.7	2.7	2.2	4.1	4.0	3.5	3.2	2.8	4.3	3.7	1.3 (23)
1esl	54–59 (6)	84.2	2.8	1.0	2.2	2.4	2.1	1.8	1.8	2.6	1.5	1.5 (1)
1rsy	74–79 (6)	88.9	2.8	0.8	4.1	3.5	6.6	3.9	4.0	3.0	3.1	2.9 (19)
1tml	45–50 (6)	77.7	3.0	2.3	6.3	5.1	4.8	3.2	5.4	4.7	5.1	2.7 (30)
1f3g	123–131 (9)	88.3	3.0	2.4	3.8	2.9	4.5	3.5	4.0	3.5	3.3	3.2 (2)
1a49A	463–472 (10)	70.5	3.3	0.9	3.9	4.9	8.1	3.8	5.0	2.3	2.7	1.6 (24)
1csn	216–223 (8)	89.2	3.0	2.6	8.1	8.9	8.7	6.3	6.5	8.8	6.1	5.8 (6)
1ialA	32–42 (11)	78.3	4.0	2.6	4.5	5.1	6.4	7.6	6.8	4.6	4.0	3.6 (23)
1df6A	10–15 (6)	70.8	4.0	2.0	3.2	3.3	3.3	3.4	3.2	3.1	3.2	3.1 (12)
1kbt	26–31 (6)	70.8	2.8	2.4	3.8	3.8	4.5	4.2	3.9	3.4	3.5	2.9 (27)
1aszA	313–322 (10)	73.6	4.8	2.1	4.4	2.4	4.6	1.8	2.9	2.3	1.9	1.7 (2)
1lxa	102–107 (6)	71.8	5.3	2.7	3.2	4.3	4.7	5.5	3.6	3.8	4.7	3.7 (23)
**Average**		**81.4**	**2.8**	**1.8**	**4.2**	**4.0**	**4.5**	**4.0**	**3.9**	**3.9**	**3.7**	**2.7 (15.8)**
**Std. dev.**		**7.0**	**1.0**	**0.6**	**1.9**	**1.6**	**1.7**	**1.7**	**1.5**	**1.6**	**1.4**	**1.2 (8.7)**

1) GDT-TS [1] of the initial template-based model

2) The all-atom RMSD of the environment is as defined in Results and Discussion.

3) All-atom RMSDs of 3 residues before and 3 residues after the loop region

4) The RMSD is calculated as root-mean-square deviation of the main-chain atoms N, C_{α}, C, and O.

5) Loop RMSD of the initial model obtained by MODELLER [2]

6) Results of the lowest-energy model structures obtained by PS-knowledge, the same procedure as GalaxyLoop-PS2 but using the energy function mainly composed of statistical energy terms

7) Results of the lowest-energy model structures obtained by PS-physics, the same procedure as GalaxyLoop-PS2 but using the energy function composed of only physics-based energy terms

8) Results of the lowest-energy model obtained by the loop refinement algorithm of ModLoop of MODELLER [3,4] are reported. From the same initial template-based model, 500 independent optimizations were performed at the refinement level ‘slow’ (in contrast to the default option of the ModLoop ‘server’ that performs 300 optimizations).

9) Results of the best-score models sampled by Next-generation KIC (NGK) using the protocol provided by Stein *et al*. [5]. 500 models were generated for each target as in Stein et al. The Rosetta program v3.5 was used.

10) Results of the lowest-energy model structures obtained by GalaxyLoop-PS1

11) Results of the lowest-energy model structures obtained by GalaxyLoop-PS2

12) RMSDs of the lowest-RMSD model structures and their energy ranks in the final bank

[1] Mariani V, Kiefer F, Schmidt T, Haas J, Schwede T (2011) Assessment of template based protein structure predictions in CASP9. Proteins 79 Suppl 10: 37-58.

[2] Sali A, Blundell TL (1993) Comparative Protein Modeling by Satisfaction of Spatial Restraints. Journal of Molecular Biology 234: 779-815.

[3] Fiser A, Do RKG, Sali A (2000) Modeling of loops in protein structures. Protein Science 9: 1753-1773.

[4] Fiser A, Sali A (2003) ModLoop: automated modeling of loops in protein structures. Bioinformatics 19: 2500-2501.

[5] Stein A, Kortemme T (2013) Improvements to robotics-inspired conformational sampling in rosetta. PLoS One 8: e63090.
