# Supplementary material for: Protein Loop Modeling Using a New Hybrid Energy Function and Its Application to Modeling in Inaccurate Structural Environments
Source: PLoS One. 2014 Nov 24;9(11):e113811. doi: 10.1371/journal.pone.0113811 (PMC4242723; doi:10.1371/journal.pone.0113811)
Supplement: Table S7 — Loop reconstruction results and modeling results on perturbed crystal structures for the 12-residue loop Set 1 using energy functions composed of either knowledge-based or physics-based energy components. (PDF) [file pone.0113811.s008.pdf]

Table S7. Loop reconstruction results and modeling results on perturbed crystal structures for the 12-residue loop Set 1 using energy functions composed of either knowledge-based or physics-based energy components.

PDB ID	Native framework RMSD (Å)[1)]		Side chain–perturbed set RMSD (Å)[1)]			Backbone-perturbed set RMSD (Å)[1)]		
	PS-knowledge[2)]	PS-physics[3)]	E-RMSD (Å)[4)]	PS-knowledge[2)]	PS-physics[3)]	E-RMSD (Å)[4)]	PS-knowledge[2)]	PS-physics[3)]
1a8d	0.4	3.0	0.7	0.5	4.3	1.6	1.2	4.3
1arb	1.3	1.1	0.9	2.1	1.1	2.3	1.5	1.4
1bhe	2.1	0.7	0.8	0.8	2.5	1.2	3.1	1.1
1bn8	0.6	4.7	0.7	0.9	1.1	3.4	1.1	5.2
1c5e	2.2	0.5	0.7	2.1	2.2	1.5	1.5	1.2
1cb0	0.5	0.9	0.8	0.6	1.4	2.7	0.8	1.1
1cnv	4.2	2.5	0.7	4.5	6.6	3.0	6.6	6.4
1cs6	1.9	2.0	1.1	3.3	3.0	1.8	1.5	3.8
1dqz	3.4	6.2	1.2	2.9	2.9	1.9	3.2	7.5
1exm	0.8	3.4	0.8	2.5	2.7	1.5	1.7	4.0
1f46	1.5	2.0	0.9	1.4	3.9	1.6	4.4	4.1
1i7p	0.4	0.6	0.9	0.7	5.0	3.3	1.6	1.5
1m3s	5.8	5.8	1.1	6.0	5.4	1.5	3.2	4.3
1ms9	1.7	1.5	0.8	2.4	1.8	1.6	1.8	1.8
1my7	2.1	0.6	1.5	3.1	1.8	4.0	1.0	2.2
1oth	0.6	0.5	1.1	2.3	3.3	1.2	0.9	0.7
1oyc	0.6	2.0	1.2	2.1	2.1	2.5	0.6	0.7
1qlw	1.1	2.0	0.6	4.9	5.0	2.3	2.0	2.3
1t1d	1.3	1.5	1.3	1.2	2.3	4.0	1.2	1.3
2pia	0.7	1.0	1.1	0.8	2.3	1.2	0.8	0.8
**Average**	**1.7**	**2.1**	**1.0**	**2.3**	**3.0**	**2.2**	**2.0**	**2.8**
**Std. dev.**	**1.4**	**1.7**	**0.2**	**1.5**	**1.5**	**0.9**	**1.4**	**2.0**

1) RMSD is calculated as the root-mean-square deviation of the main-chain atoms N, C_α, C, and O.

2) Results of the lowest-energy model structures obtained by the same procedure as GalaxyLoop-PS2 but using the energy function mainly composed of knowledge-based energy terms, named here as PS-knowledge. The weight parameters are set to ($w_{\text{Electrostatics}}$, w_{SA}, $w_{\phi/\psi}$, w_χ, w_{Hbond}, $w_{\text{atom-pair}}$) = (0.0, 0.0, 1.2, 1.0, 4.0, 12.0).

3) Results of the lowest-energy model structures obtained by the same procedure as GalaxyLoop-PS2 but using the energy function composed of only physical energy terms, named here as PS-physics. The weight parameters are set to ($w_{\text{Electrostatics}}$, w_{SA}, $w_{\phi/\psi}$, w_χ, w_{Hbond}, $w_{\text{atom-pair}}$) = (0.25, 0.02, 0.0, 0.0, 0.0, 0.0).

4) The all-atom RMSD of the environment is as defined in Results and Discussion.
