# Supplementary material for: Protein Loop Modeling Using a New Hybrid Energy Function and Its Application to Modeling in Inaccurate Structural Environments
Source: PLoS One. 2014 Nov 24;9(11):e113811. doi: 10.1371/journal.pone.0113811 (PMC4242723; doi:10.1371/journal.pone.0113811)
Supplement: Table S8 — Loop modeling results for the 12-residue loop Set 1 in three different environments with a smaller number of CSA bank size ( N = 30 instead of N = 50). (PDF) [file pone.0113811.s009.pdf]

Table S8. Loop modeling results for the 12-residue loop Set 1 in three different environments with a smaller number of CSA bank size (N = 30 instead of N = 50).

PDB ID	Native framework RMSD (Å)[1)]		Side chain–perturbed set RMSD (Å)[1)]			Backbone-perturbed set RMSD (Å)[1)]		
	Galaxy PS2 bank30[2)]	Best sampled (rank)[3)]	E-RMSD (Å)[4)]	Galaxy PS2 bank30[2)]	Best sampled (rank)[3)]	E-RMSD (Å)[4)]	Galaxy PS2 bank30[2)]	Best sampled (rank)[3)]
1a8d	3.1	1.3 (3)	0.7	0.6	0.6 (1)	1.6	2.2	2.1 (10)
1arb	1.1	1.1 (1)	0.9	1.4	1.4 (1)	2.3	1.9	1.3 (4)
1bhe	1.3	1.3 (1)	0.8	2.1	1.0 (6)	1.2	3.2	2.2 (9)
1bn8	1.4	0.8 (4)	0.7	1.5	1.1 (3)	3.4	1.2	1.1 (8)
1c5e	2.0	0.8 (3)	0.7	2.0	0.6 (24)	1.5	2.5	1.0 (2)
1cb0	0.5	0.5 (1)	0.8	0.5	0.5 (1)	2.7	0.9	0.9 (4)
1cnv	2.4	2.1 (9)	0.7	2.4	2.4 (1)	3.0	6.5	5.6 (10)
1cs6	3.6	1.7 (7)	1.1	2.9	2.6 (20)	1.8	1.5	1.5 (2)
1dqz	0.9	0.9 (1)	1.2	6.2	3.7 (12)	1.9	6.1	2.0 (12)
1exm	4.0	2.8 (22)	0.8	3.0	1.5 (12)	1.5	1.6	1.5 (3)
1f46	2.0	1.7 (16)	0.9	1.9	1.6 (5)	1.6	2.8	2.8 (18)
1i7p	0.3	0.3 (1)	0.9	0.5	0.5 (1)	3.3	4.1	1.8 (3)
1m3s	6.0	4.8 (26)	1.1	5.5	4.1 (26)	1.5	6.0	2.2 (19)
1ms9	1.7	0.9 (17)	0.8	1.6	0.8 (11)	1.6	1.8	1.0 (27)
1my7	0.5	0.5 (1)	1.5	1.0	1.0 (1)	4.0	2.4	0.9 (2)
1oth	0.5	0.5 (2)	1.1	2.3	1.8 (2)	1.2	0.8	0.8 (1)
1oyc	2.1	1.6 (7)	1.2	1.1	1.1 (1)	2.5	0.9	0.9 (1)
1qlw	4.8	2.7 (8)	0.6	4.1	3.3 (30)	2.3	1.4	1.4 (1)
1t1d	2.6	1.5 (2)	1.3	2.4	1.1 (18)	4.0	2.5	2.1 (3)
2pia	0.7	0.7 (1)	1.1	0.8	0.8 (1)	1.2	0.8	0.8 (1)
**Average**	**2.1**	**1.4 (6.7)**	**1.0**	**2.2**	**1.6 (8.9)**	**2.2**	**2.6**	**1.7 (7.0)**
**Std. dev.**	**1.5**	**1.0 (7.5)**	**0.2**	**1.5**	**1.1 (9.5)**	**0.9**	**1.7**	**1.0 (7.1)**

1) RMSD is calculated as the root-mean-square deviation of the main-chain atoms N, C_α, C, and O.

2) Results of the lowest-energy model structures obtained by the same procedure as GalaxyLoop-PS2 but using a smaller number of CSA bank members (N = 30)

3) RMSDs of the lowest-RMSD model structures and their energy ranks in the final bank

4) The all-atom RMSD of the environment is as defined in Results and Discussion.
