# Supplementary material for: Protein Loop Modeling Using a New Hybrid Energy Function and Its Application to Modeling in Inaccurate Structural Environments
Source: PLoS One. 2014 Nov 24;9(11):e113811. doi: 10.1371/journal.pone.0113811 (PMC4242723; doi:10.1371/journal.pone.0113811)
Supplement: Table S9 — Contribution of each energy component. (PDF) [file pone.0113811.s010.pdf]

Table S9. Contribution of each energy component.

Energy component	E_{Coulomb}	$E_{\text{FACTS,GB}}$	$E_{\text{FACTS,SA}}$	$E_{\phi/\psi}$	E_{χ}	E_{Hbond}	$E_{\text{atom-pair}}$
Weight[1)]	0.16	0.16	0.05	1.2	1.0	4.0	12.0
Contribution (%)[2)]	7.6	9.6	6.8	19.9	10.0	7.9	38.1

1) Energy weight used for each component

2) Contribution is defined as the standard deviation of each energy term for the training set decoy conformations averaged over training loop target multiplied by the energy weight. These values are then normalized.
