# Supplementary material for: Protein Loop Modeling Using a New Hybrid Energy Function and Its Application to Modeling in Inaccurate Structural Environments
Source: PLoS One. 2014 Nov 24;9(11):e113811. doi: 10.1371/journal.pone.0113811 (PMC4242723; doi:10.1371/journal.pone.0113811)
Supplement: Table S10 — Correlation between energy and decoy loop RMSD calculated using different subsets of energy components. (PDF) [file pone.0113811.s011.pdf]

Table S10. Correlation between energy and decoy loop RMSD calculated using different subsets of energy components.

Energy function subset										Correlation[2]
Component	E_{bonded}	E_{vdW}	$E_{Coulomb}$	$E_{FACTS,GB}$	$E_{FACTS,SA}$	$E_{\phi/\psi}$	E_{χ}	E_{Hbond}	$E_{atom-pair}$	
	1.0	1.0	-	-	-	-	-	-	-	0.03
	-	-	0.16	0.16	-	-	-	-	-	0.10
	-	-	-	-	0.05	-	-	-	-	0.12
	-	-	0.16	0.16	0.05	-	-	-	-	0.22
	-	-	-	-	-	-	-	-	12.0	0.52
	-	-	-	-	-	1.2	-	-	12.0	0.48
Weight combinations[1]							1.0	-	12.0	0.52
	-	-	-	-	-	-	-	4.0	12.0	0.48
	-	-	-	-	-	1.2	1.0	4.0	12.0	0.47
	-	-	0.16	0.16	-				12.0	0.52
	-	-	0.16	0.16	-	-	1.0	-	12.0	0.52
	-	-	0.16	0.16	0.05	-	1.0	-	12.0	0.50
	-	-	0.16	0.16	0.05	1.2	1.0	4.0	12.0	0.46

1) Energy weight used for each component to generate different subsets of the energy components

2) Pearson correlation coefficient calculated between the training set decoy loop Cα RMSD and energy and averaged over the training set loops
