# Supplementary material for: Protein Loop Modeling Using a New Hybrid Energy Function and Its Application to Modeling in Inaccurate Structural Environments
Source: PLoS One. 2014 Nov 24;9(11):e113811. doi: 10.1371/journal.pone.0113811 (PMC4242723; doi:10.1371/journal.pone.0113811)
Supplement: Text S1 — Detailed information on Methods and Results. (PDF) [file pone.0113811.s012.pdf]

Text S1. Detailed information on Methods and Results.

Implementation of FACTS solvation free energy in GALAXY

FACTS [1], a type of Generalized Born/Surface Area (GB/SA) solvation free energy, is the most computationally expensive term of all the GalaxyLoop-PS2 energy components. However, no approximations are made to increase computational efficiency. The effective Born radii and their gradients, for which computation is the most costly, are calculated at every evaluation of energy to maximize the performance of the local minimizer L-BFGS-b [2]. Instead, the computational efficiency was improved by avoiding redundant calculations of GB pair interactions. Lists of atom pairs with changed effective Born radii upon loop conformational change are updated during energy optimization and the GB pair interaction energy is re-evaluated only for those pairs. The relative permittivity values for the protein interior and solvent are set to 1 and 78.5, respectively.

The χ angle preference term

The side-chain χ angle energy term is expressed as a sum of residue contributions $E_{\mathrm{rot}}^{(k)}$ derived from the Dunbrack backbone-dependent rotamer library [3], where the contribution $E_{\mathrm{rot}}^{(k)}$ of the k-th residue is expressed as follows:

$$E_{\mathrm{rot}}^{(k)}\left(\left\{\chi_j^{(k)}\right\}\right) = -\log\left[\sum_{i=1}^{N_{\mathrm{rot}}^{(k)}} p_i^{(k)} F_i^{(k)}\left(\left\{\chi_j^{(k)}\right\}\right)\right],$$

$$F_i^{(k)}\left(\left\{\chi_j^{(k)}\right\}\right) = \frac{1}{\left(2\pi\right)^{n^{(k)}/2} \Pi_j^{n^{(k)}} \sigma_{ij}^{(k)}} \exp\left[-\frac{1}{2}\sum_{j=1}^{n^{(k)}}\left(\frac{\chi_j^{(k)} - \overline{\chi}_{ij}^{(k)}}{\sigma_{ij}^{(k)}}\right)^2\right],$$

where $\chi_j^{(k)}$ is the j-th χ angle value of the k-th residue at the current protein conformation, $N_{\mathrm{rot}}^{(k)}$ is the number of rotamers in the library for the k-th residue (*e.g.*, 3 for Leu, 81 for Arg), $p_i^{(k)}$ is the probability for the i-th rotamer of the k-th residue, $n^{(k)}$ is the number of χ angles in the k-th residue (*e.g.*, 1 for Leu, 4 for Arg), and $\bar{\chi}_{ij}^{(k)}$ and $\sigma_{ij}^{(k)}$ are the mean and standard deviation of the i-th rotamer of the k-th residue provided in the library.

Derivation of the ϕ/ψ preference term

The ϕ/ψ preference energy term was derived after determining weights for all the other terms, so as to correct the bias in the secondary structure of the overall energy function. In the initial energy function, a ϕ/ψ preference term was included, obtained by taking a minus logarithm of the standard Ramachandran maps (for three residue types PRO, GLY, and others in 10°-angle bins). Next, new Ramachandran maps representing the ϕ/ψ-angle preference of the models for the training set generated with the initial energy function were obtained. Bias-corrected Ramachandran maps were then calculated by subtracting the new maps from the standard maps, and the ϕ/ψ preference energy term was updated using these bias-corrected maps. This procedure of correcting bias in the Ramachandran maps was repeated three times. The energy function and gradients were calculated by bicubic interpolation [4].

Contribution of each energy component

Contributions of 7 out of the 9 energy components to the total energy were estimated by the standard

deviations of the energy values of the individual components for the training set decoy conformations. The two energy terms E_{bonded} and E_{vdW} were not considered because these terms are important for maintaining protein-like geometry, and decoy conformations with poor geometry can introduce too much energy fluctuation. As can be seen in **Table S9**, contribution of the knowledge-based atom-pair potential dipolar-DFIRE [5] is the largest while those of other energy components are rather similar. The dipolar-DFIRE term also shows the highest correlation between energy and decoy loop RMSD (See **Table S10**). Addition of different combinations of physics-based energy terms (E_{Coulomb}, $E_{\text{FACTS,GB}}$, and $E_{\text{FACTS,SA}}$) or other knowledge-based energy terms tend to decrease the correlation coefficient. However, importance of energy components cannot be solely determined by the correlation coefficient. For instance, E_{bonded} and E_{vdW} show very low correlation of 0.03 but are essential for maintaining protein-like geometry. According to our experiences with loop modeling, additional terms are also important for generating conformations with physically accurate charge interactions, hydrogen bonds, etc. Although we do not show that the current combination of energy components constitute the best scoring function by running loop modeling calculations for all possible combinations of energy components, it is shown that the combination of physics-based energy and knowledge-based energy is superior to either physics-based energy or knowledge-based energy in the subsection “Comparison of the hybrid energy with the physics-based and knowledge-based energy” of the main text.

[1] Haberthur U, Caflisch A (2008) FACTS: Fast analytical continuum treatment of solvation. Journal of Computational Chemistry 29: 701-715.

[2] Liu DC, Nocedal J (1989) On the Limited Memory Bfgs Method for Large-Scale Optimization. Mathematical Programming 45: 503-528.

[3] Dunbrack RL, Cohen FE (1997) Bayesian statistical analysis of protein side-chain rotamer preferences. Protein Science 6: 1661-1681.

[4] Keys RG (1981) Cubic Convolution Interpolation for Digital Image-Processing. Ieee Transactions on Acoustics Speech and Signal Processing 29: 1153-1160.

[5] Yang YD, Zhou YQ (2008) Specific interactions for ab initio folding of protein terminal regions with secondary structures. Proteins 72: 793-803.
